# Letter to the Editor: Fibroblast activation protein and the CaMKIIδ–Calcineurin–NFAT pathway in diabetic HFpEF

**DOI:** 10.1042/CS20258366

**Published:** 2026-01-09

**Authors:** Xinyu Nie, Xingyue Feng, Can Xu

**Affiliations:** 1 Department of Cardiac Surgery, Nanjing Drum Tower Hospital, The Affiliated Hospital of Nanjing University Medical School, Nanjing, 210008, China

**Keywords:** fibroblast activation protein, heart failure with preserved ejection fraction, type 2 diabetes mellitus, cardiac fibrosis', calcium signaling

## To the Editor,

Li et al. have provided important new evidence that fibroblast activation protein (FAP) directly contributes to the pathogenesis of diabetic heart failure with preserved ejection fraction (HFpEF) by engaging the CaMKIIδ–Calcineurin–NFATc2 pathway [1]. Their experiments showed that genetic deficiency or pharmacological inhibition of FAP not only improved diastolic function but also reduced myocardial fibrosis, oxidative stress, and inflammatory activation. What stands out is the mechanistic bridge they draw between fibroblast activity and calcium-dependent transcriptional remodeling—an area that, until now, has not been a central focus in HFpEF research.

The clinical angle is straightforward. In their models, inhibition of FAP with Talabostat restored relaxation and preserved energetic balance [1]. That contrasts with agents such as semaglutide which, in the Semaglutide Treatment Effect in People with obesity (STEP)-HFpEF Diabetes Mellitus (DM) trial, improved symptoms and exercise capacity largely through weight loss and systemic anti-inflammatory effects [2]. Put differently, current therapies address metabolic and hemodynamic contributors but leave the fibrotic substrate of HFpEF largely untouched. Because impaired relaxation and fibrosis define the syndrome, targeting FAP offers a genuinely complementary strategy. The notion of combining metabolic modulators with anti-fibrotic intervention should be tested directly, and circulating FAP activity could be evaluated as a biomarker within multiparametric frameworks (e.g.,the Heart Failure Association Pre-test assessment, Echocardiography & natriuretic peptide, Functional testing, Final aetiology [HFA-PEFF] score and the Heavy, Hypertensive, Atrial Fibrillation, Pulmonary Hypertension, Elder, Filling Pressure [H₂FPEF] score ) to capture remodeling biology not reflected in current scores.

Translation need not be limited to small molecules. Preclinical work using anti-FAP chimeric antigen receptor T cells shows that selective elimination of activated fibroblasts can suppress cardiac fibrosis [3]. Together with pharmacologic inhibition, this broadens the therapeutic toolbox and opens the door to stage- and phenotype-specific strategies. The practical hurdles are familiar: define target selectivity and safety, and establish pharmacodynamic markers that track on-target cardiac effects in patients.

It is helpful to place these findings within the prevailing HFpEF paradigm. The influential framework proposed by Paulus and Tschöpe centers on comorbidity-driven endothelial inflammation and microvascular dysfunction as upstream drivers of impaired energetics [4]. That model remains instructive but incomplete. By positioning FAP upstream of CaMKIIδ–Calcineurin–NFATc2 signaling, Li et al. add a tractable axis that connects fibroblast activity to maladaptive calcium signaling in cardiomyocytes. Their observation that FAP deficiency enhances AMPK phosphorylation also hints at a metabolism–calcium–fibrosis triad, a construct that could reconcile the clinical heterogeneity of HFpEF across diabetic, obese-predominant, and hypertensive endotypes [1].

Parallels from oncology reinforce biological plausibility. FAP-positive cancer-associated fibroblasts orchestrate matrix remodeling and immune evasion and are now targets of active therapeutic development [5]. The fact that the same stromal program drives both tumor progression and cardiac fibrosis encourages cross-disciplinary learning and strengthens the rationale for advancing FAP-directed therapies in cardiovascular disease.

In our view, the priorities are clear. First, incorporate FAP activity into HFpEF phenotyping and risk stratification to capture the remodeling dimension that present tools miss. Second, prospectively identify FAP-sensitive endotypes beyond type 2 diabetes mellitus and test rational combinations that pair FAP inhibition with established metabolic agents. Third, accelerate therapeutic development along two tracks—more selective inhibitors and biomarker-supported immune-based strategies—supported by biomarkers and, where feasible, non-invasive imaging of cardiac FAP activity. Pursuing these steps could help the field move from describing HFpEF to intervening on it with precision, while acknowledging that validation in well-phenotyped clinical cohorts will be essential for translation.

## Open Access

This article has been published open access under our Subscribe to Open programme, made possible through the support of our subscribing institutions, learn more here: https://portlandpress.com/pages/open_access_options_and_prices#conditional

## Abbreviations

FAP: fibroblast activation protein
HFpEF: heart failure with preserved ejection fraction
